# From Fax to Secure File Transfer Protocol: The 25-Year Evolution of Real-Time Syndromic Surveillance in England

**DOI:** 10.2196/58704

**Published:** 2024-09-17

**Authors:** Alex J Elliot, Helen E Hughes, Sally E Harcourt, Sue Smith, Paul Loveridge, Roger A Morbey, Amardeep Bains, Obaghe Edeghere, Natalia R Jones, Daniel Todkill, Gillian E Smith

**Affiliations:** 1 Real-time Syndromic Surveillance Team UK Health Security Agency Birmingham United Kingdom; 2 NIHR Health Protection Research Unit in Emergency Preparedness and Response King's College London London United Kingdom; 3 School of Environmental Sciences University of East Anglia Norwich United Kingdom

**Keywords:** epidemiology, population surveillance, sentinel surveillance, public health surveillance, bioterrorism, mass gathering, pandemics

## Abstract

The purpose of syndromic surveillance is to provide early warning of public health incidents, real-time situational awareness during incidents and emergencies, and reassurance of the lack of impact on the population, particularly during mass gatherings. The United Kingdom Health Security Agency (UKHSA) currently coordinates a real-time syndromic surveillance service that encompasses 6 national syndromic surveillance systems reporting on daily health care usage across England. Each working day, UKHSA analyzes syndromic data from over 200,000 daily patient encounters with the National Health Service, monitoring over 140 unique syndromic indicators, risk assessing over 50 daily statistical exceedances, and taking and recommending public health action on these daily. This English syndromic surveillance service had its origins as a small exploratory pilot in a single region of England in 1999 involving a new pilot telehealth service, initially reporting only on “cold or flu” calls. This pilot showed the value of syndromic surveillance in England, providing advanced warning of the start of seasonal influenza activity over existing laboratory-based surveillance systems. Since this initial pilot, a program of real-time syndromic surveillance has evolved from the single-system, -region, -indicator pilot (using manual data transfer methods) to an all-hazard, multisystem, automated national service. The suite of systems now monitors a wide range of syndromes, from acute respiratory illness to diarrhea to cardiac conditions, and is widely used in routine public health surveillance and for monitoring seasonal respiratory disease and incidents such as the COVID-19 pandemic. Here, we describe the 25-year evolution of the English syndromic surveillance system, focusing on the expansion and improvements in data sources and data management, the technological and digital enablers, and novel methods of data analytics and visualization.

## Introduction

The early 2000s saw the dawn of a new era of public health threat: bioterrorism. In the United States, the September 11, 2001, attacks and anthrax mail incidents underlined a heightened terrorist threat [1,2]. The particular emphasis on the potential for the deliberate and malicious release of highly pathogenic organisms (as highlighted by the anthrax mail incident) raised awareness and concern around bioterrorism to new levels. In the United States, a novel form of public health surveillance had emerged, with an increasing focus on providing timely sources of health intelligence to strengthen monitoring of the health impact of potential bioterror attacks. This form of surveillance was termed “syndromic” as the data capture systems underpinning it primarily focused on monitoring the presentation of chief complaints, symptoms, or provisional diagnoses presented to health care providers, rather than laboratory-confirmed cases of the disease [3-5]. Syndromic surveillance can now be defined as “the (near) real-time collection, analysis, interpretation and dissemination of health-related data to enable the early identification of the impact (or absence of impact) of potential human public-health threats which require effective public health action” [6].

The authors of this viewpoint paper share a combined work experience of over 150 years coordinating syndromic surveillance in England. Here we look back over the last 25 years and reflect on the evolution of syndromic surveillance in England, “from fax to SFTP (secure file transfer protocol).” We outline the journey that English syndromic surveillance has taken with respect to changes in technology, methodology, drivers and enablers, and epidemiology.

## Syndromic Surveillance in England: The Early Years

In England, syndromic surveillance first started in the late 1990s as a small, opportunistic project, led by a single public health epidemiologist working in a regional field epidemiology team in the West Midlands. At the time, there had been a focus on sentinel general practitioner (GP; family physicians available to patients generally during working hours) network surveillance, which, while not classically considered “syndromic,” is now considered to be part of the “syndromic toolkit.”

Within the United Kingdom, the National Health Service (NHS) is a publicly funded health care system that provides free health care services at the point of use for most residents. In 1998, a new NHS telephone health helpline (NHS Direct) was being piloted across parts of England [7,8]. A number of factors converged to start the English syndromic surveillance journey: a regional director of public health with an innovative approach to public health and a public health epidemiologist with an interest in novel surveillance were working together in the West Midlands, one of the regions where the new telehealth system was being piloted. The following question was asked: “can NHS Direct call data be used for influenza surveillance to improve timeliness over existing influenza surveillance systems?”

The answer and outcome of this project was “yes”; the telehealth data showed promise for public health surveillance [9]. While NHS Direct calls specific to patients with presenting “cold or flu” symptoms were higher at times when known influenza was circulating, the timing of the increase in activity was in advance of the increase in traditional (laboratory-based) reporting for influenza [10,11]. In England, this provided a “lightbulb moment” for syndromic surveillance and the benefits it could bring to the practice of public health surveillance. It was the initial impetus for developing a national program of work over the next 2 decades.

## The Major Drivers for Development

As a new and relatively unknown form of public health surveillance at that time, the early development of syndromic surveillance in England initially struggled to attract resources and support for expansion. However, over the last 2 decades, several drivers have highlighted the importance, benefits, and ongoing need for syndromic surveillance. These drivers often involved major public health “incidents” (where an unexpected organism, environmental, or other hazard has been identified but the aim is to assess the hazard and risk and prevent impact on health, eg, an outbreak) where syndromic surveillance was used to support the public health response, thereby highlighting its impact in the response to public health emergencies.

As previously described, syndromic surveillance in England was initially conceived during the late 1990s. However, its use in major public health incidents and “events” (a predictable future situation that is likely to have an impact on the health of the population, eg, a mass gathering) have strengthened confidence in and understanding of the use of syndromic surveillance, enabling the development and continuous improvement of a suite of syndromic surveillance systems over the decades.

In 2005, a large explosion and fire (Buncefield Oil Depot, Hertfordshire, United Kingdom) posed a significant and very visible threat to public health [12]. Sizeable parts of South-East England (including the Greater London urban conurbation) were exposed to a large smoke plume with a potential for air pollution causing harm to the population. At the time, the near real-time monitoring of NHS Direct telehealth calls and GP consultations were used to assess changes in health care–seeking behaviors in areas affected by the smoke plume or increases in presentations of syndromes potentially linked to smoke exposure, for example, breathing difficulty and asthma [13]. The findings from the regular analysis of data from these syndromic surveillance systems and the use of the data in the dynamic risk assessments all provided reassurance of the lack of impact on the public’s health to the national incident management team. This incident demonstrated the use of syndromic time-series data in providing trend and historical context (the observed over expected) and near real-time (daily) delivery and analysis (here of daily telehealth data) to support a major incident response.

Another major early driver in the evolution of the English syndromic surveillance service was the 2009 influenza pandemic. At this time, England’s Health Protection Agency (HPA) operated 2 national syndromic surveillance systems: the continuing NHS Direct telehealth system and a new expanded GP consultation surveillance system. In addition to insights from laboratory, hospital, and mortality surveillance, syndromic surveillance monitored a set of relevant indicators that provided intelligence into how the pandemic was progressing. Syndromic surveillance was able to identify and monitor very early local activity, which was initially focused in urban and deprived districts of Birmingham, in the West Midlands region of England, before syndromic surveillance then subsequently monitored the wider spread of disease throughout the country [14].

One of the biggest drivers for the expansion of the English syndromic surveillance service was the London 2012 Olympic and Paralympic Games. In total, 2 new national syndromic surveillance systems were commissioned to provide daily data on health care–seeking trends of the population: the Emergency Department Syndromic Surveillance System (EDSSS) and the GP out-of-hours system [15,16]. A new bespoke statistical exceedance algorithm was developed to provide automated statistical alarms on syndromic data [17]. A novel risk assessment process was developed and implemented to standardize the review of syndromic exceedances and other key epidemiological signals, facilitate timely and consistent decision making including timely alerting of partners, and support better documentation of decision-making [18]. Underpinning this development program were increased resources, in the form of additional people in the team to deliver the new developments, and money to resource the expansion of new systems. A key legacy of the Games was securing a recurring increased budget to maintain the new developments, systems, ways of working, and analytical methods. The London 2012 enhanced syndromic surveillance response became “business as usual” going forward, thus providing a greater ability to support routine and seasonal surveillance of key public health threats [15].

Other major events have helped demonstrate the use and importance of syndromic surveillance in the public health system. These have included the public health response to an ash cloud from an Icelandic volcanic eruption [19]; population health monitoring during the 2022 Commonwealth Games; geopolitical events, for example, the G8 Summit [20]; the COVID-19 pandemic [21,22]; and assessments of the health impact of air pollution [23].

## The Evolution of Syndromic Surveillance Systems

In England, syndromic surveillance systems have been traditionally developed at a country level rather than at the region or state level. This is possible due to the advantage of having a NHS that is freely accessible by the whole population at the point of need. The national syndromic surveillance service in England has evolved from a single system with one source of data to 6 national real-time systems with several data sources.

**Figure 1:**
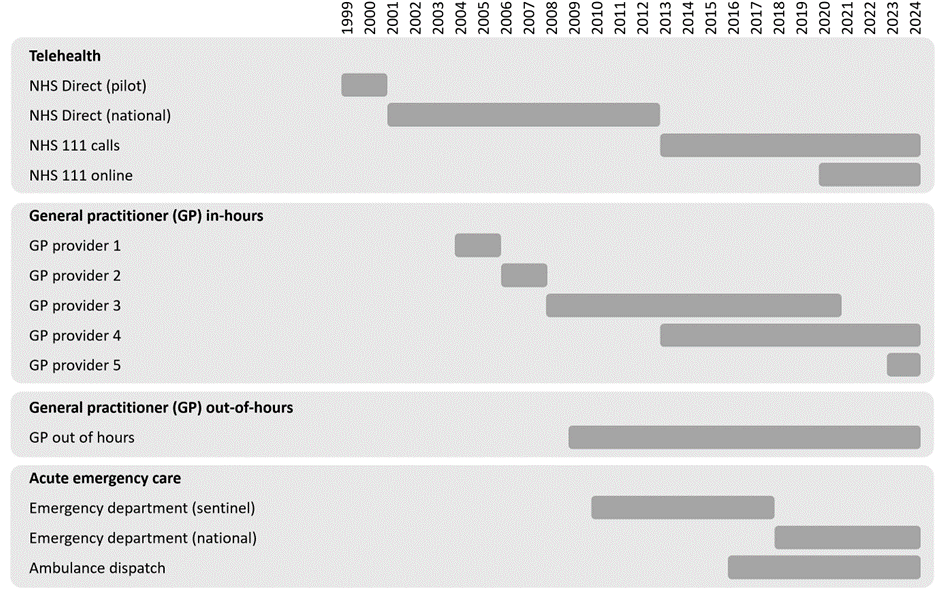
The evolution of syndromic surveillance systems and the underlying data streams used in the English syndromic surveillance system, 1999-2024. GP: general practitioner; NHS: National Health Service.

However, one limitation of this approach is that syndromic surveillance systems are vulnerable to changes within the NHS. The adoption of different clinical patient management systems, organizational changes, improvements to clinical guidance, and updates to clinical systems can all affect the data collected and its availability for syndromic surveillance purposes. In the last 25 years there have been multiple major changes to the syndromic surveillance systems used in England and some of the data sources underpinning those systems. Altogether, there have been 13 different incarnations of the systems and their evolving data providers (Figure 1).

The NHS Direct national telehealth service ran for over 10 years but was replaced by a new, free-to-access telehealth service, but in 2013 was replaced by a new, free to access telehealth service, NHS 111. On the face of it, syndromic surveillance outputs spanning NHS Direct and NHS 111 have remained largely similar, reporting on a selection of syndromic indicators, for example, cold or flu and cough [24]. However, the logistical work in moving between different telehealth services was substantial, requiring engagement with different stakeholders, governance requirements, data sharing agreements, data architecture and pathways, reporting tools, and adjustment of statistical baselines.

As NHS patient services evolve, syndromic surveillance systems are flexible and can capture new and potentially useful sources of health care data. An example is the NHS 111 online service, which launched nationally in 2018 as an alternative point of access for nonurgent care support [25]. This provided a unique opportunity for syndromic surveillance to capture a novel set of digital data from patients potentially not using other established NHS services. The NHS 111 online syndromic surveillance system was launched during the COVID-19 pandemic to bolster situational awareness and now supports day-to-day surveillance of both infectious and noninfectious hazards [26].

Another significant change over the years was the EDSSS. This started as a small sentinel emergency department (ED) surveillance network of approximately 30 EDs across England [15]. Initial pilot work was completed in 2011 in time for its use during the London 2012 Olympic and Paralympic Games [15]. The development of EDSSS was a close collaboration between the HPA and the Royal College of Emergency Medicine, the national professional body for emergency medicine in the United kingdom. EDSSS provided benefits for both partners: HPA developed the first ED syndromic surveillance system in England to contribute to its public health surveillance function, and the Royal College of Emergency Medicine was able to pilot a national clinical minimum dataset among EDs that participated in the EDSSS. This work provided proof of principle that the code-set used for recording clinical information could be standardized nationally [15]. The early sentinel EDSSS provided the basis for the development of the national Emergency Care Data Set, which has improved data capture and reporting across EDs in England [27]. The Emergency Care Data Set provided an opportunity to revolutionize ED syndromic surveillance in England, with the fundamental gain of EDSSS changing from a small sentinel system to a near-national system, collecting data from 150 EDs across England [28,29].

GP syndromic surveillance has also evolved. The aim of this surveillance is to monitor trends in the consulting behavior of patients using the underlying clinical codes entered into the patient record (by the GP) to track consultation rates among the registered population. In England, there are several different providers of GP electronic patient management systems. The GP syndromic surveillance system has evolved from using data from a single provider to now using data from multiple providers.

Another notable advancement during the last 25 years has been the adoption of electronic patient records across the NHS. The vast majority of patient attendances are now recorded electronically, removing the need for paper-based records to be digitalized retrospectively. This facilitates real-time surveillance as the record is immediately available for interrogation. Furthermore, the coding of patient episodes has improved over the years, as standardized coding systems have been introduced. In England, SNOMED CT (Systematized Medical Nomenclature for Medicine–Clinical Terminology) is now the NHS-wide clinical coding standard used across primary and secondary care services [30]. The result of this for syndromic surveillance is the harmonization of code lists across systems and the ability to develop expertise and knowledge of a single coding system.

The availability of electronic patient records also allows for the inclusion of more complex and varied data fields within the syndromic dataset. In England, the syndromic data extracted from patient records has expanded and evolved. In the early years, simple counts of patient episodes with a syndromic code were extracted. Now, much more detailed and granular information (still retaining anonymity) can be routinely extracted or derived, including (1) severity measures, (2) treatments, (3) ethnicity, (4) investigations, and (5) measures of deprivation [22,31,32]. These additional fields can be vital during investigations of potential threats, incidents, or events, allowing more information to feed into the epidemiological assessment of the situation, which can provide further insights into the characteristics of the affected population, allowing for improved targeted actions to be taken.

One consequence of the continual evolution and expansion of the current United Kingdom Health Security Agency (UKHSA) syndromic surveillance program is that data volumes have increased substantially. In the beginning, a pilot system, in one area of England, resulted in a small selection of indicators being analyzed each week. Now, each day, UKHSA analyzes syndromic data from over 200,000 daily NHS patient encounters, monitoring over 140 unique syndromic indicators, risk assessing more than 50 daily statistical exceedances, and alerting and taking public health action on those each day. This has required an advancement in data analytics methods to manage the expansion of data and the number of factors under investigation. Early methods included statistical process control charts and standardized incidence ratios to determine the unusual activity that needed further investigation. The bespoke statistical methods developed for the London 2012 Olympic and Paralympic Games are used in the live daily syndromic system today. This statistical method was developed to provide a single robust approach for all syndromic surveillance systems, including any future new data sources. It is sophisticated enough to automatically adjust for changes in data provider volumes, amend scale accordingly (from national to local signals), incorporate day-of-the-week effects, including public holidays, and detect both spikes and significant changes compared with previous years [17].

A further consequence of changing system data feeds (eg, due to operational or contractual reasons; Figure 1) is the challenge of analyzing data from different sources over the longer term. Following a transition to a different syndromic data feed in a syndromic surveillance system, it is possible to compare trends over the time periods covering the 2 different feeds; however, changes in service provision at the new health care provider, different coding, or patient-presenting behaviors can make comparisons challenging. Statistical techniques can be adopted to “correct” the new data to the reporting levels of the old source; however, this also comes with difficulties and risks. Furthermore, over the 25-year span of the UKHSA syndromic surveillance program, health care services, patient health care–seeking behavior, and the general health of the population have changed significantly over time. These factors also make comparisons of contemporary and historical data challenging, as baseline levels will be different, and it is therefore difficult to interpret data and draw conclusions.

## The Evolution of Technology

The early syndromic surveillance developments in England, including small-scale sentinel GP surveillance and the pilot of the NHS Direct telehealth call system, were underpinned by technologically crude data transfer methods. The surveillance of NHS Direct call data was initially established based on the faxing of weekly reports from the data provider. Each faxed report contained the number of calls for a small number of key syndromic indicators, for example, cold or flu. Data were manually entered from the physical fax into a spreadsheet. Before the advent of “big data” and “cloud-based” technologies, faxes were a standard method for securely sharing data between the data provider and the public health agency.

In England, the first advances in syndromic data-sharing technology came through routine emailing of syndromic data. At first, this continued as a manual process requiring human intervention to both send and receive the email; however, eventually, this process was adapted to use automated email procedures, removing the need for human involvement in the process. Asking busy NHS services to manually email syndromic data reports each day was not sustainable. Very quickly, automation became a standard for establishing syndromic surveillance systems in England. The subsequent development of automated processes for importing data into a database at the public health organization also removed manual resource requirements and the risk of transcription errors from the manual process.

During the 2000s, emailing of data evolved into more sophisticated transfer methods, including SFTP, only possible as the storage of data evolved from workbooks to databases. More recently, new NHS-based secure data transfer methods, for example, Message Exchange for Social Care and Health, have once again revolutionized the technology underpinning syndromic surveillance [33]. Overall, each of these technological advancements has made it possible to transfer larger and more complex sets of data more securely and reliably.

This increase in data volumes and complexity has driven the need for bigger and more efficient data storage solutions. Therefore, there has developed a need for expanded data storage capacity. During the early years, syndromic data storage consisted of Excel worksheets (Microsoft Corporation). However, as the number of systems increased and historical data accumulated, these systems quickly outgrew the capacity that Excel could provide. SQL database platforms became the standard for storing and interrogating data. To this day, SQL databases feed other investigation and statistical analysis tools that are used for analyzing and visualizing data. Similarly, in the early years, data analysis and visualization were exclusively limited to Excel. However, this has evolved, and now a suite of different data analytics tools is used for analysis and visualization. Open access tools, such as R Studio (Posit team), provide a wide range of analytical and statistical tools with the ability to deliver outputs and visualizations. In UKHSA, syndromic surveillance data are now processed using SQL for storage, R Studio for statistical exceedances, and a combination of Microsoft Excel, R Studio, and Power BI (Microsoft Corporation) for interrogating data through dashboards and other digital tools.

In England, the format for reporting and disseminating syndromic surveillance data and intelligence is one area that has remained relatively unchanged as other surveillance stages have evolved. The early years saw the development of static reports, published in PDF format, containing a collection of surveillance data charts, key messages, caveats and limitations, and background information to the syndromic system [34]. This approach remained largely the same to this day [35]. However, the COVID-19 pandemic saw an appetite for disseminating public health surveillance data and intelligence through dashboards, and for UKHSA, this is becoming the standard approach to sharing and reporting surveillance insights and intelligence. This provides a more dynamic user-friendly platform with the ability to customize data interrogation and reports and extract underlying data. For UKHSA syndromic surveillance, this is also the future direction, and plans are underway to integrate key syndromic outputs into the UKHSA data dashboard [36].

## Syndromic Collaboration

Over the last 25 years, collaboration has been vital in the successful development and expansion of real-time syndromic surveillance in England. It cannot be understated how important collaboration with data provider organizations has been to the successful evolution of our syndromic surveillance service. Often, these relationships and collaborations are built not on contractual or transactional relationships, and purchasing of data, but on a shared understanding of the importance of the work and the public health benefit and impact. Several of the provider collaborations in England have been established now for 10-15 years. During this time, relationships, understanding, and trust are built, which are essential for a positive and fruitful collaboration and successful syndromic surveillance system. This factor is often overlooked in favor of a focus on data and technology, but without the “people,” the best data and technology platforms do not automatically mean success [37].

Outside of the data provider relationships, the English syndromic surveillance service has benefited from a valuable network of international collaborators who have helped shape the program. The International Society for Disease Surveillance operated from 2005 to 2019, providing excellent contacts for the English syndromic program and linking the team with a range of technical and expert syndromic support networks. The annual International Society for Disease Surveillance conference provided a platform for collaboration, sharing innovation and experience, and showcasing excellence [38].

In Europe, the wider development and implementation of syndromic surveillance has been less common across individual countries compared with the growth across individual states in the United States. France, however, was an early European innovator in syndromic surveillance, providing a blueprint from which the English ED syndromic surveillance was developed. The 2015 European-wide “Triple-S” project provided a framework for syndromic surveillance in Europe, which led to a collaborative approach to coordinating syndromic surveillance across the continent [6]. Despite Triple-S providing an impetus for developing syndromic surveillance across Europe, the further development of a European-wide syndromic surveillance network could not take advantage of the momentum of Triple-S due to a lack of funding. However, an informal cross-country collaborative Euro-syndromic network remains a legacy of Triple-S, with cross-country collaboration across several European countries and the US Centers for Disease Control and Prevention [23,39].

Closer to home, syndromic work across the United Kingdom’s devolved administrations (England, Scotland, Wales, and Northern Ireland) has been productive over the years. Joint working has delivered cross-border projects and strengthened the UK response to events such as large mass gatherings [20,39]. There are continuing UK collaborations to share knowledge and harmonize syndromic approaches and systems to provide a better UK-focused syndromic approach in the future.

## The Next 25 Years

Over the course of the professional careers of the authors, syndromic surveillance in England has evolved from the exploration of a novel surveillance pilot project to a mainstay source of national public health intelligence. It has developed from an innovative source of data examining seasonal influenza surveillance only to an all-hazard surveillance service, supporting a wide range of infectious and noncommunicable disease surveillance programs.

However, syndromic surveillance cannot “sit on its laurels” and stay still. Over the next 25 years, syndromic surveillance must move with the times and continue to evolve at pace to tackle future emerging health threats. Syndromic surveillance needs to continue to innovate and take advantage of new advances in technology and analytical methodologies. Importantly, this evolution must also prioritize the “human element” to develop and train the next generation of leaders and experts in syndromic surveillance to drive forward these future programs.

One key priority must be the standardization and harmonization of systems across countries and continents. Currently, different countries operate different suites of syndromic surveillance systems, even where there are similarities between countries with respect to the type of surveillance undertaken. For example, within ED syndromic surveillance systems, although the general concept of an ED may broadly be the same in all locations (providing acute emergency care), there are disparities in the syndromes monitored (which may be due to the availability of the clinical information and coding formats used); the methods used for analysis and interpretation of results; and the outputs delivered to both public health professionals, health care providers in general, as well as to the public. While there will always be a level of autonomy required across each country, there are opportunities to develop minimum and common datasets to aid the interpretation of syndromic data across countries rather than between countries.

Delivering such harmonization will require the strengthening of international networks and the formation of a network of excellence, with those leading exponents of syndromic surveillance enabling knowledge mobilization and delivering plans for strengthening harmonization. However, such networks require resources, support, and dedication to deliver a structured program of development. There is also a role for syndromic surveillance in supporting the model of a national biosurveillance network, which has collaborative surveillance at its heart, given its multisource and multisectoral surveillance approach [40]. Existing international networks, such as the World Health Organization (WHO) Epidemic Intelligence from Open Sources initiative, may also play a future role in fostering these collaborative networks and nurturing technological and methodological advances [41].

Syndromic surveillance is on a new wave of technology-driven growth, which will almost certainly be underpinned by cloud-based storage and machine learning among others. While each of these represents an opportunity to improve surveillance, we must not get distracted from the basics of epidemiological surveillance and analysis and the fundamental aims and objectives of the surveillance we undertake.

Data protection, security, and governance have also played an influencing role in syndromic surveillance over the last 2 decades. On reflection, during the early stages of syndromic surveillance, there was a lower “threshold” of control around the use of patient data for public health research and surveillance. However, in more modern times, there is a wider understanding of the ethics and risks (but also the benefits) of using patient data, and as such, the governance framework has been strengthened around surveillance systems and public health organizations as a whole. In the general population, this has also led to a far greater understanding about how an individual’s health data are used. The controls and restrictions around the use of patient (and particularly identifiable) data are now far stricter. While this direction of travel has been a necessary and positive move, it does mean that for syndromic surveillance (where anonymized health record data are used the majority of the time), it is far more difficult and complex to access the health data that underpin the systems.

One final future aspiration of UKHSA syndromic surveillance is to support and facilitate the expansion of syndromic surveillance in resource-poor countries. This is still an area where syndromic surveillance in general has poor uptake, likely due to multiple factors, such as resource constraints issues, underdeveloped health and public health systems, and limited capacity and capability to establish and maintain syndromic surveillance systems. There are future opportunities to engage in knowledge exchange with other countries and expand work already ongoing to spread the benefits of syndromic surveillance globally [42,43].

## Summary

Over the last 25 years, the English national syndromic surveillance program has evolved from a small pilot project to a national real-time multisystem all-hazard program. This journey started with an innovative public health doctor (GES) taking a local idea, which, thanks to the initial collaboration with local public health experts, has grown into a national surveillance program with global partnership. It has been an exemplar of how opportunity, chance, innovation, technology, and public health expertise can come together to materialize into a world-leading service, supporting complex and varied public health incidents and events.

## Abbreviations

ED: emergency department
EDSSS: Emergency Department Syndromic Surveillance System
GP: general practitioner
HPA: Health Protection Agency
NHS: National Health Service
SFTP: secure file transfer protocol
SNOMED CT: Systematized Nomenclature of Medicine–Clinical Terms
UKHSA: United Kingdom Health Security Agency
WHO: World Health Organization
